# Evaluation of *Saccharomyces cerevisiae* Wine Yeast Competitive Fitness in Enologically Relevant Environments by Barcode Sequencing

**DOI:** 10.1534/g3.119.400743

**Published:** 2019-12-02

**Authors:** Simon A. Schmidt, Radka Kolouchova, Angus H. Forgan, Anthony R. Borneman

**Affiliations:** * The Australian Wine Research Institute, PO Box 197, Glen Osmond, South Australia, Australia, 5064 and; † Department of Genetics and Evolution, University of Adelaide, South Australia, Australia, 5000

**Keywords:** Competitive growth, barcode sequencing, bar-seq, copper tolerance, sulfite tolerance

## Abstract

When a wine yeast is inoculated into grape juice the potential variation in juice composition that confronts it is huge. Assessing the performance characteristics of the many commercially available wine yeasts in the many possible grape juice compositions is a daunting task. To this end we have developed a barcoded *Saccharomyces cerevisiae* wine yeast collection to facilitate the task of performance assessment that will contribute to a broader understanding of genotype-phenotype relations. Barcode sequencing of mixed populations is used to monitor strain abundance in different grape juices and grape juice-like environments. Choice of DNA extraction method is shown to affect strain-specific barcode count in this highly related set of *S. cerevisiae* strains; however, the analytical approach is shown to be robust toward strain dependent variation in DNA extraction efficiency. Of the 38 unique compositional variables assessed, resistance to copper and SO_2_ are found to be dominant discriminatory factors in wine yeast performance. Finally, a comparison of competitive fitness profile with performance in single inoculum fermentations reveal strain dependent correspondence of yeast performance using these two different approaches.

The ability to dominate and efficiently ferment the compositionally variable medium of grape juice are fundamental properties of yeasts used in the wine industry. These properties are ultimately dependent on overcoming a range of environmental variables, including nitrogen content (Beltran *et al.* 2005; Crépin *et al.* 2012) oxygen availability (Sablayrolles *et al.* 1996; Salmon 2006; Varela *et al.* 2012), juice clarity (Delfini and Costa 1993; Casalta *et al.* 2016), micronutrient concentration (Kudo *et al.* 1998; Schmidt *et al.* 2011) and vitamin availability (Medina *et al.* 2012). A host of factors can influence the composition of juice, including annual climate variation (Hagen *et al.* 2008), vineyard location (Roullier-Gall *et al.* 2014), vineyard management (Neilsen *et al.* 2010) and grape processing (Delfini 1993; Bindon *et al.* 2013; McRae *et al.* 2015; Day *et al.* 2019). These factors not only affect chemical composition, but also microbial ecology of grape juice (Hierro *et al.* 2006; Bokulich *et al.* 2014; Zarraonaindia *et al.* 2015; Morrison-Whittle and Goddard 2018; Lleixà *et al.* 2018). The multitude of possible variations in juice composition combined with the large number of available commercial yeast strains has, by necessity, limited the types of association studies that could be conducted.

Assessment of large numbers of yeast strain *x* environmental conditions have predominantly involved screening on solid agar (Tong *et al.* 2001) or in miniaturized liquid culture (Warringer and Blomberg 2003; Liccioli *et al.* 2011; Piotrowski *et al.* 2017), but larger formats have also been described (Peltier *et al.* 2018). However, the development of parallel DNA sequencing technology has made possible competitive fitness profiling of mixed populations across multiple samples (Smith *et al.* 2010). Furthermore, the development of specific analytical approaches (Robinson *et al.* 2014; Simpkins *et al.* 2019) and novel methods of barcode introduction (Roy *et al.* 2018) has seen several studies using mixed populations to explore the relationship between yeast and environment (Cubillos *et al.* 2009; Han *et al.* 2010; Gresham *et al.* 2011; Sideri *et al.* 2014; Payen *et al.* 2016; Piotrowski *et al.* 2017; Maclean *et al.* 2017).

In this work we describe the development of a barcoded collection of wine-related *Saccharomyces cerevisiae* strains. This collection is comprised of commercial strains, historical strains from the Australian Wine Research Institute (AWRI) culture collection and winery isolates that were selected to represent the broader genetic diversity of *S. cerevisiae* yeasts used in the wine industry. The impact of extraction conditions on biases in strain representation within mixed pools is explored. Finally, the fitness of 82 wine-related *S. cerevisiae* strains in different oenologically relevant defined media and grape juice compositions is presented.

## Methods

### Yeast strains and culturing

The strains used in this work were selected to be representative of the different clades spanning the full spectrum of natural genetic variation within wine yeasts. The full list of yeast strains used is provided in File_S1-T1. All of these strains have been sequenced and the details of their relationship to other wine yeasts has been previously published (Borneman *et al.* 2016). All strains were obtained from The Australian Wine Research Institute (AWRI) Culture Collection. Strains were maintained on YPD agar (1% w/v yeast extract, 2% w/v peptone and 2% w/v D-Glucose).

Pools of barcoded yeast (barcoding described below) were produced by growing individual strains overnight in 10 ml YPD. The optical density of each strain was recorded to ensure similar representation of each strain in the subsequent pool (File_S1-T4). The overnight cultures were mixed in a sterile beaker, the old medium was removed by centrifugation (1000 × g for 10 min) and resuspended in fresh YPD to an absorbance at 600 nm (OD600) of 20.48. To this was added an equal volume of sterile 40% glycerol to give a final OD600 of 8.5. 10 ml aliquots were dispensed into yellow capped tubes and frozen at -80° to be used as inoculum into competitive fitness experiments at an inoculation rate of 1mL / 100mL of medium (WYBC pool).

**Figure 1:**
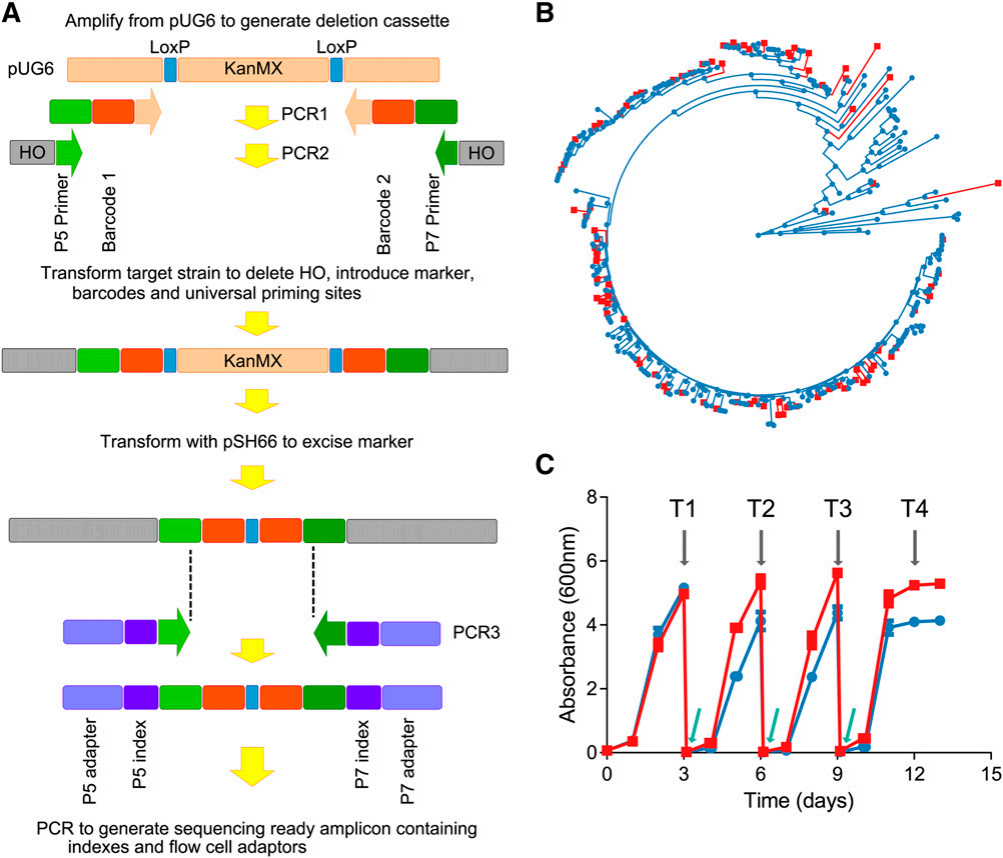
Scheme for generation and analysis of barcoded wine yeast collection (A). (B) Maximum-likelihood phylogeny of 236 yeast strains as described in Borneman *et al.* (2016) with barcoded strains shown in red. C) Typical growth profile of pooled yeast during a serial batch experiment. Growth in defined medium containing either 200 g/L (▪) or 280 g/L (●) 50:50 glucose/fructose was determined by absorbance at 600 nm. Gray arrows show sampling times and green arrows show re-inoculation. Data points represent mean ± SD (n = 3) for both treatments.

Competitive fitness experiments were conducted in defined medium the composition of which is described in Schmidt *et al.* (2011) or grape juice. Grape juice was obtained from freshly pressed grapes and either used immediately or stored frozen at -20°. Experiments were performed in 100 mL vessels with a sample port and directional-flow check valve to release CO_2_. The vessels were stirred at 350 rpm and incubated at 18°. Standard-condition defined medium was inoculated directly with 1 ml of freshly thawed WYBC pool. For experiments L1 – L6 pooled strains were inoculated into the reference medium and grown until they reached the end of exponential growth. For experiments L7 and L8 involving fresh grape juice pooled strains were inoculated directly into reference and treatment media. Once cultures has reached stationary phase 1 mL was taken from each flask and used to inoculate subsequent control and treatment flasks in a serial batch mode. Sequential inoculation was performed twice more for a total of 4 time points for each experiment. All treatments were performed in triplicate. The scheme for the serial batch ferments and sampling times relative to absorbance is shown in Figure 1C. Growth profiles as measured by absorbance at 600 nm, and sugar concentration during the growth phase, is shown for all treatments in File_S2.

### Juice and defined medium composition analysis

Glucose and fructose concentrations were determined enzymatically (Hohorst 1965) with adaptations as described by Vermeir *et al.* (2007) for performance of 100-μl assays in 96-well microtiter plates. Other compositional analyses were undertaken by The Australian Wine Research Institute (AWRI) Analytical Service [International Organization for Standardization 17025 accredited laboratory, Adelaide, SA, Australia]. Metal ion concentrations were determined as described by Wheal *et al.* (2011) on a Perkin-Elmer (Waltham, USA) inductively coupled mass spectrometer model Nexion 350D with the following settings: RF power 1400W, plasma argon flow rate 18 L/min, nebulizer flow rate 0.75-0.80 L/min. Yeast assimilable nitrogen (YAN) was determined using a combination of the NOPA assay for determination of free amino nitrogen (Dukes and Butzke 1998) and enzymatic determination of ammonium. YAN was calculated as follows: YAN (mg/L) = ammonium × 0.825 (mg/L) + Free amino Nitrogen (FAN, mg/L). SO_2_ concentration was determined by the aspiration method (Rankine and Pocock 1970).

### Introducing barcodes into wine yeast

A scheme for the introduction of molecular barcodes into yeast is shown in Figure 1A. A transformation cassette was generated in two stages. Initially a marker gene (*KanMX*) flanked by two LoxP sites was amplified from a plasmid (pUG6 – Euroscarf, (Güldener *et al.* 1996)) using primers pUG6_GBC#_F and pUG6_GBC#_R where # is a number between 1 and 109 (Figure 1A). All primers are listed in File_S1-T2. These primers contain a pUG6 (EuroScarf) homologous sequence, a short molecular barcode and part of the Illumina P5 and P7 sequencing primers respectively. The barcode sequences used were derived from (Caporaso *et al.* 2012) and are listed in File_S1-T1. Combinations of barcode 1 and barcode 2 allowed the unique molecular tagging of up to 96 strains with a total of 20 primer pairs. PCR conditions were; 95° for 1 min and 10 cycles of 95° for 10 s, 61° for 15 s, 72° for 45 s. The product generated from the above PCR was then used as template in a second PCR reaction that primed from the P5 and P7 sequences using primers HOssExt_IllumSeq_Fv6 and HOssExt_IllumSeq_Rv6 (File_S1-T2). These primers introduced 79 and 78 base sequences with homology to *HO* (YDL227C) into the product. Conditions for this PCR were 95° for 1 min and 29 cycles of 95° for 10 s, 55° for 15 s, 72° for 90 s. Selected wine yeast strains were transformed with this product using an adaptation of the LiAc method described by Gietz *et al.* (1992). Transformants were isolated on YPD plates containing 200 μg/ml G418. Transformation led to the replacement of 1539 bases internal to *HO* from base 128 – 1666 (relative to ATG) with the cassette (Figure 1A).

Transformants were restreaked on YPD:G418 and the fidelity of the insertion within the *HO* ORF was verified at both ends by PCR using primers KanB + HO_Conf1 and KanMX_U21 + CVHO_R respectively (File_S1-T2). Verified barcode-containing strains were transformed with the Cre-expressing plasmid PSH66 (EuroScarf, (Hegemann and Heick 2011)). Positive transformants were identified through selection on 200 μg/ml G418 + 100 μg/ml ClonNat (WERNER BioAgents GmbH) on YPD to select for strains carrying both the *KanMX* cassette and the plasmid PSH66 respectively. Colonies resistant to both G418 and ClonNat were picked onto YPD:Gal (20 g/L):ClonNat (50ug/ml) and incubated at 28° for 3 days to induce Cre-mediated excision of the *KanMX* cassette (Figure 1A). ClonNat resistant colonies were patch plated onto YPD:G418 and YPD without selection to identify transformants that had lost the *KanMX* marker. Loss of *KanMX* was confirmed in these strains by PCR using HO_Conf1 and CVHO_R primers (File_S1-T2). Finally, strains for which excision of *KanMX* had been confirmed were cured of pSH66 by passage through YPD without selection and screening of passaged strains on YPD:ClonNat to ensure loss of ClonNat resistance. Each barcoded strain was then sequenced across the barcode region to verify the barcode for each strain. The sequence of each strains barcode insert is provided in File_S1-T3. An alignment showing the sequences across the barcoded region of each strain is presented in File_S2, Figure S11. This process generated transformants from which amplicons suitable for paired-end Illumina-based sequencing could be generated using a single PCR reaction (see below).

### Generation of amplicons from mixed culture

Genomic DNA was extracted using a Gentra Puregene Yeast/Bact kit (Qiagen) according to the manufacturer’s instructions except that 6 μl of lytic enzyme was used. The transformation procedure described above resulted in the replacement of *HO* with a pair of 12 bp barcodes separated by a loxP scar and flanked by primer sites homologous to those used to initiate priming on Illumina sequencing platforms (Figure 1A). Sequence-ready amplicons were generated by PCR using primers Illum_P5_S50(1-8) + Illum_P7_N70(1-12) (File_S1-T2) containing P5 and P7 indexes respectively, thereby permitting molecular dual-indexing for up to 96 experimental conditions. 50ng of genomic DNA was used as template and amplified using the following conditions; 95° for 1 min and 25 cycles of 95° for 10 s, 64° for 5 s, 72° for 30 s.

### Sample preparation and sequencing

PCR product concentrations were determined using a Qubit fluorometer (ThermoFischer scientific). 300ng of PCR product from each sample point was added to a pool. The pooled amplicon was cleaned using a NucleoSpin PCR cleanup kit (Macherey-Nagel) and the eluted product brought to a concentration of 5 ng/μl. Sequencing was performed on an Illumina HiSeq 2500 in rapid run mode using a 150 bp single ended, dual indexing kit by the Ramaciotti Centre for Genomics (University of New South Wales, Sydney, Australia). Raw sequence data were trimmed to remove primer sequences using cutAdapt (v1.8.1), with processed reads mapped to a library of barcode sequences (File_S1-T3) using Blat (Kent 2002). We refer to the number of sequences mapped to a barcode as the barcode count.

### Statistical analysis

The strain resolved barcode counts for each indexed sample were used as raw input for the determination of changes in yeast strain representation within the mixed population. Statistical evaluation was done using EdgeR (Robinson *et al.* 2010) in R version 3.3.2 (R Core Team 2016). Briefly, data were filtered to remove samples with little or no information (*i.e.*, samples with fewer than 15 reads and strains with fewer than 3 reads). Libraries were RLE normalized using the method of Anders and Huber (2010). A nested pairwise design was constructed according to the following formula; ∼0 + treatment + treatment:time. Where fresh juices were used the formula ∼ treatment + treatment:time was implemented with the reference condition as the intercept. Dispersions were estimated using a Generalized Linear Model (GLM) approach and the model fitted using a quasi-likelihood GLM (glmQLFit()). Comparisons between pairs of treatments were made using glmQLFTest. Data analysis and graphical representation were further aided using the package ggplot2 (Wickham 2009). Hierarchical clustering of fitness data were performed using the eclust function from the factoExtra package in R with hc_metric = “euclidean” and hc_method = “ward.D2”. The heat map was generated using the pheatmap package (v 1.0.12). The function ‘ggpairs’ from GGally package was used to evaluate correlations in reference conditions across batches (method = spearman). The complete code used for these analyses is made available in File_S3.

The reference condition in this work is growth of yeasts in a defined medium constructed to reflect the composition of a Chardonnay juice with minimal trace elements as previously described (Schmidt *et al.* 2011). The same reference medium was used in 6 of the 7 experiments comprising the data set presented here, with the exception being experiment L7 in which a Chardonnay juice was used as a reference condition. Yeast strain fitness in the reference condition was evaluated as the contrast between strain-specific barcode count after 2 serial propagations relative to the strain-specific barcode count after a single growth phase (T3 control – T1 control). Strain fitness in a test condition was evaluated as the contrast between strain-specific barcode count in the test condition over time relative to strain-specific barcode count in the reference condition over time ((T3 test - T1 test) - (T3 control – T1 control)). A full list of all test conditions, their abbreviations, description and respective reference conditions is provided in File_S1-T5 and File_S1-T6. Results from all reported pairwise comparisons are provided in File_S1-T7. Graphs showing growth and sugar utilization during all pooled inoculum serial batch experiments is provided in File_S2.

A relationship between optical density and cell number was established using *S. cerevisiae* strain EC1118 grown under semi-anaerobic conditions in defined medium. Cell numbers were estimated by plating of diluted cell cultures on YPD agar. Absorbance of cell cultures were determined (600 nm) at the time of plating. The equation Log_10_(cell number) = 1.011 × log_10_(abs) + 7.489 was derived and used to estimate cell numbers when necessary (Files_S2, Figure S9).

### Data availability

Materials produced in this study are available upon request. The authors affirm that all data necessary for confirming the conclusions of this article are represented fully within the article, its figures and supplementary materials. File_S1-T1 contains a list of strains and sequences of barcodes introduced into each strain. File_S1-T2 contains a primer list. File_S1-T3 contains complete sequence of the 150 bp barcode element inserted into each strain. File_S1-T4 provides the absorbance of individual cultures prior to pooling. File_S1-T5 and File_S1-T6 contains a summary of experimental conditions. File_S1-T7 provides the results of all pair-wise comparisons (log2 fold-change) and associated statistics. File_S2 includes all supplementary figures including absorbance and sugar utilization data for all experiments discussed in the manuscript. File_S3 provides the code used in pair wise comparison of strain performance. All raw count data are provided in File_S4. Supplemental material is available at figshare. Supplemental material available at figshare: https://doi.org/10.25387/g3.10565996.

## Results and Discussion

### Creation of a representative barcoded wine yeast set

The vast majority of strains of the yeast *S. cerevisiae* that are considered suitable for industrial wine production are represented by a large single clade within the larger *S. cerevisiae* species phylogeny (Borneman *et al.* 2016). In order to provide a collection of barcoded strains that would broadly represent this *S. cerevisiae* wine clade, a subset of 94 wine yeast strains, consisting primarily of wine-clade isolates, were chosen from a larger, previously sequenced set of strains such that even minor genetic clades would be represented by at least one member. These strains are listed in File_S1-T1 and highlighted in red in Figure 1B.

A unique bipartite molecular barcode was then inserted into the open reading frame of *HO* in each of the 94 strains to be investigated, thereby enabling the unambiguous measurement of individual strain abundance within a mixed population of strains (Figure 1A). The two halves of each barcode were initially separated by a G418 resistance gene (*KanMX*), which was subsequently removed via Cre-mediated excision. This reaction generated the final 150 bp genomic element containing two 12 bp barcodes (separated by a 92 bp LoxP scar), flanked by Illumina sequencing compatible priming sites. By incorporating the Illumina priming sites into the genome, it was possible to generate barcode amplicons from every strain in the collection with the same primer sequence and in a single PCR reaction, thereby allowing the use of limited amplification cycles and minimizing the potential for PCR bias. Furthermore, the barcode element is sufficiently compact to allow for the inclusion of both indexes and flow cell adaptors into the amplification primers, resulting in the generation of sequencing ready amplicons in a single step.

### The conditions of extraction affect the rates of detection of individuals within a mixed population

**Figure 2:**
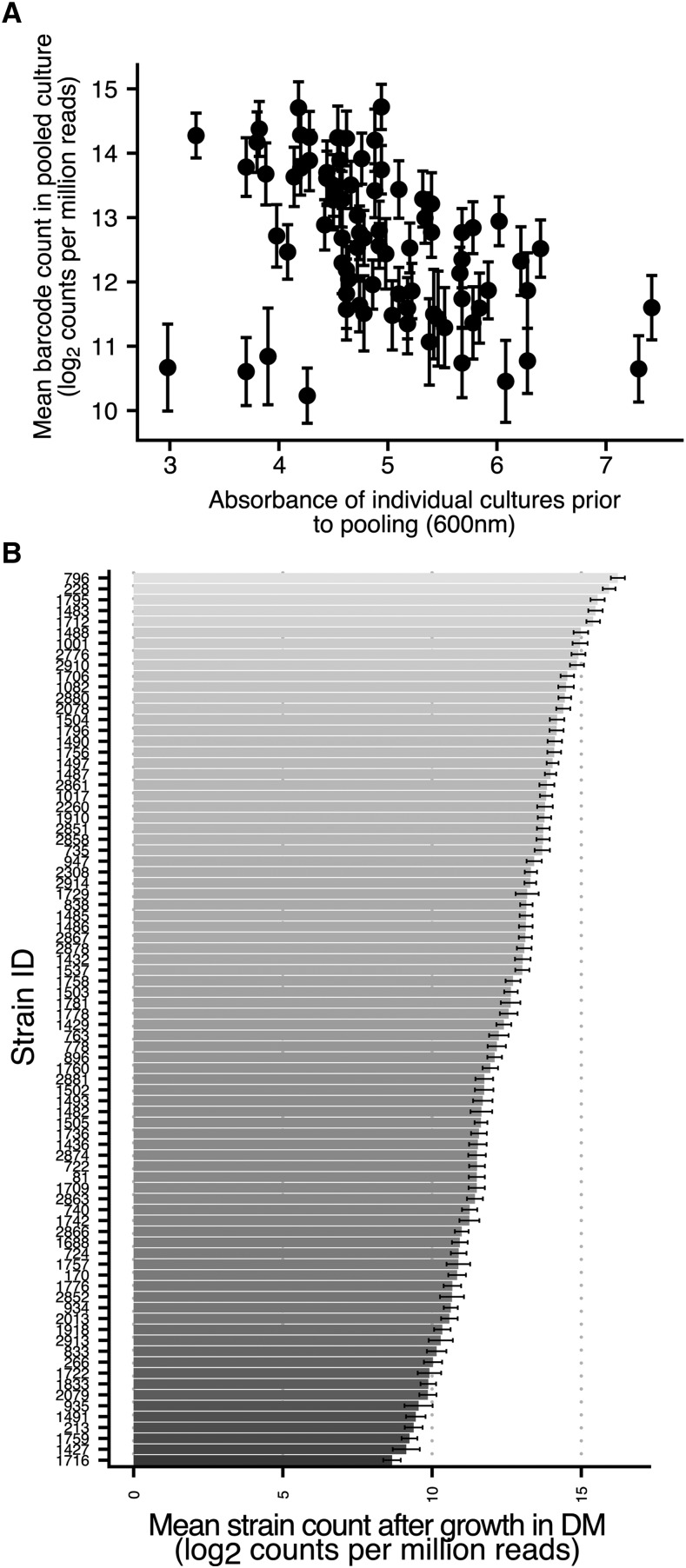
Variability and reproducibility of strain detection. (A) Strain-specific barcode count from extracts of pooled culture frozen stocks (y-axis, mean ± SD, n = 3) compared to the absorbance recorded prior to culture pooling (x-axis). (B) Barcode count per strain (mean ± SD, n = 24) after growth in defined medium (DM). Extractions and amplicon production were performed independently, barcode sequencing for each was performed in a single sequencing run.

Unlike other barcoded strain collections, that look at the effect of specific mutations within a defined genetic background, the Wine Yeast Barcode Collection is comprised of a heterogenous collection of wild-type genetic backgrounds. These different genetic backgrounds may have significant effects on growth, but also on the effectiveness of protocols for DNA extraction, which could impact on the ability to accurately study abundance using the DNA barcodes. In order to assess the later, a combined pool of 87 barcoded strains was created by mixing individual aliquots of stationary-phase liquid cultures of each barcoded wine yeast strain. DNA was then extracted from frozen aliquots of this pool and subjected to DNA extraction, barcode PCR and high-throughput sequencing. Of the 87 strains in the pool, 82 could be resolved from the sequence data. A wide distribution of barcode counts was observed following sequencing of amplicons generated from the pool, ranging more than 22-fold from 1202 ± 359 to 26615 ± 6694 (mean ± SD) (Figure 2A). Whereas cell concentrations, estimated from absorbance measures, covered only a 2.fivefold range (estimated cell density = 9.3 × 10^7^ to 2.35 × 10^8^ cfu/ml) across all 82 strains. In fact, strains that had the same absorbance prior to being added to the pool (abs 600nm = 4.9 ± 0.3) varied in their barcode counts by nearly 10-fold (2700 to 26600). Overall absorbance values of cultures prior to pooling and log transformed barcode counts were inversely correlated (pearson r = -0.396, *P* = 0.00025). This demonstrates a disconnect between absolute strain abundance within a pool and barcode count. Such a disconnect limits the use of barcode counts for comparisons of abundance between strains.

Several factors may account for the high variance in barcode count for strains with similar absorbance values. Strains of different sizes can produce different absorbance values when the same number of cells are present and this can vary with growth stage (Stevenson *et al.* 2016). Whether differences in optical density/cell concentration relationships between strains could explain the variance in barcode count was tested by evaluating the relationship for the subset of strains with mean absorbance (abs 600nm = 4.9) at the time of making the pool. These strains were grown overnight in YPD, their absorbances measured, and cell concentration determined by hemocytometer count (File_S2, Figure S12). Across a range of absorbance values from 3.8 to 4.7 the cell concentration varied by 1.sixfold (8.5 × 10^7^ to 1.4 × 10^8^). Therefore, strain specific differences in the relationship between absorbance and cell concentration cannot account for the 10-fold variance in barcode count observed for this subset of strains.

Alternatively, polymerase related amplification has been shown to influence both length and GC content of sequencing libraries (Dabney and Meyer 2012). PCR amplification from the WYBC pool initiates with the bases of the barcodes themselves, however, we have not observed enrichment of barcodes for GC content or specific terminal bases in either high count or low count amplicons. Other than the 2 × 12 bases of the barcodes themselves the structure of the amplicon is the same for each yeast strain. We therefore consider this an unlikely source of barcode count variation. Finally, individual strains may be more or less amenable to DNA extraction which may also be influenced by growth stage. If strains differ in the DNA extraction efficiency, then genetic heterogeneity of the pool is a likely source of the variation described above.

Despite the variance in barcode counts for equivalently represented strains, estimates of strain specific responses to environmental stimuli are possible if strain specific extraction is reproducible. Reproducibility of strain-specific barcode detection was assessed through analysis of an experiment in which 24 flasks of defined medium, compositionally representative of grape juice, were inoculated with the same volume of pooled inoculum. Samples were taken after three days of growth and indexed amplicons were sequenced on a single flow cell. The normalized strain-specific barcode counts are shown in Figure 2B. Despite the variation in sequence depth between strains the strain-specific barcode count was highly reproducible across 24 independent extractions with the coefficient of variation of log_2_-transformed barcode count ranging from 1.3 to 4.9%, showing that for all strains the technical variance is a small fraction of the mean.

The wide distribution of raw barcode count and low absolute barcode count of a substantial number of strains is undesirable as it results in inefficient utilization of sequencing capacity; doubling the number of reads mostly serves to increase count depth for the strains with highest counts, while the dynamic range of strain specific fitness determinations is reduced in those strains with the lowest counts. Furthermore, if variance in barcode count results from growth stage or genetic heterogeneity related differences in DNA extractability then it may be possible to overcome these differences through revisions to the extraction method.

**Figure 3:**
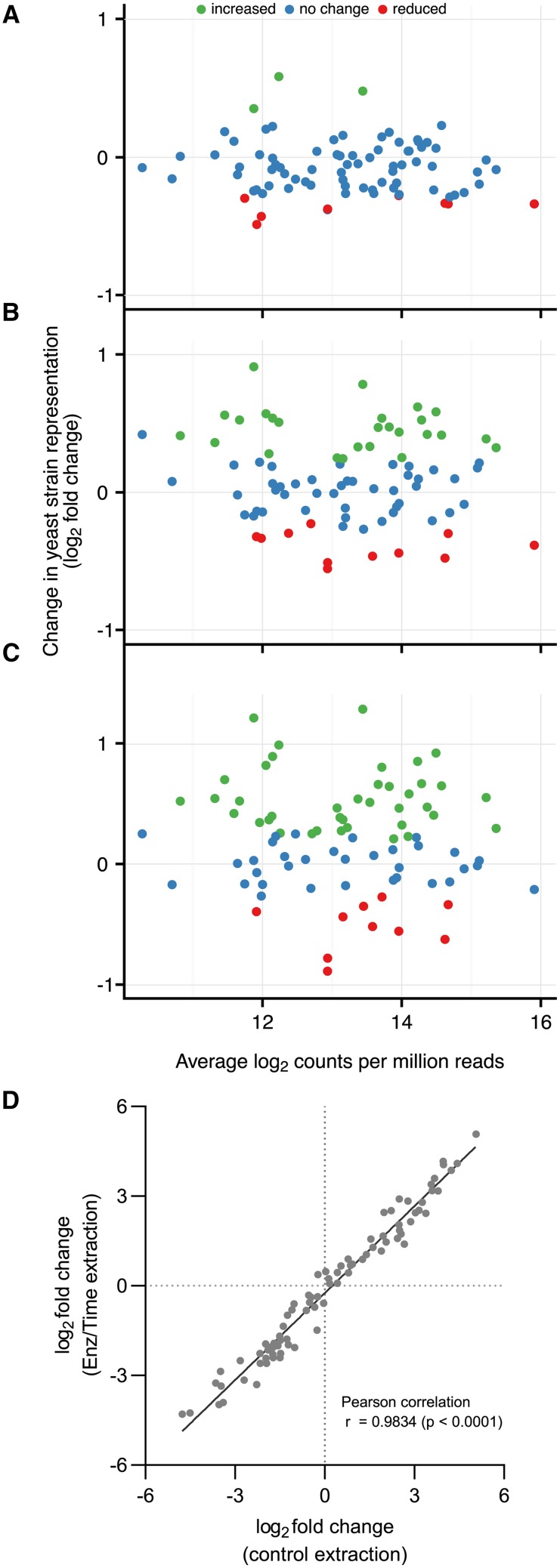
Effect DNA extraction conditions on strain detection. Three extractions conditions A) 12 ul enzyme + 40 min incubation, B) 6 ul enzyme + 80 min incubation, C) 12 ul enzyme + 80 min incubation relative to a standard extraction condition (6 ul enzyme + 40 min incubation) were evaluated and the effects on strain detection in subsequent BarSeq determined. Red and green colored spots show strains that are significantly different compared to the reference condition. D) The fitness of strains grown in defined medium relative to strains grown YPD were evaluated using either a control extraction (6 ul enzyme + 40 min incubation) or the extraction conditions used in C (12 ul enzyme + 80 min incubation). All data shows the mean log_2_ fold change from 3 independent replicates (n = 3).

Modifications to the DNA extraction protocol were evaluated in an attempt to alleviate the large strain specific barcode count distribution. A column-free extraction method utilizing an enzyme-based lysis approach was used in this work. The effect of 2 enzyme concentrations (3 and 6 times the recommended concentration), the effect of enzyme incubation duration, or both enzyme concentration and incubation duration on the distribution of raw counts was evaluated (Figure 3). The representation of strains in the data set was shown to be dependent on the DNA extraction method used prior to amplicon preparation. However, it was not only the strains in the bottom percentile of the count distribution that were affected, and for some strains with poor representation the number of counts associated with them either did not change or was made worse by increasing the concentration of lyticase used during DNA extraction. These results show that while alterations to the DNA extraction method can alter the barcode count for specific barcodes within the pool, they do not systematically alleviate the wide distribution in strain-specific barcode counts observed across the full collection of barcoded strains.

While the effect of extraction method on strain-specific barcode count across a single mixed population was strain dependent, it was unclear as to whether these differences had any effect on strain fitness estimates, which are based on relative proportions of barcode counts across two samples. Extraction-dependent differences in fitness estimates were therefore assessed through a comparison of growth of the mixed population in defined medium relative to growth in YPD, while employing two different extraction regimes (Figure 3D). Equivalent strain-specific fitness estimates were derived regardless of the extraction method used, suggesting that while absolute extraction biases could not be overcome, fitness values display an analytical robustness with respect to the DNA extraction method.

### Wine yeast strain fitness in wine relevant conditions

**Figure 4:**
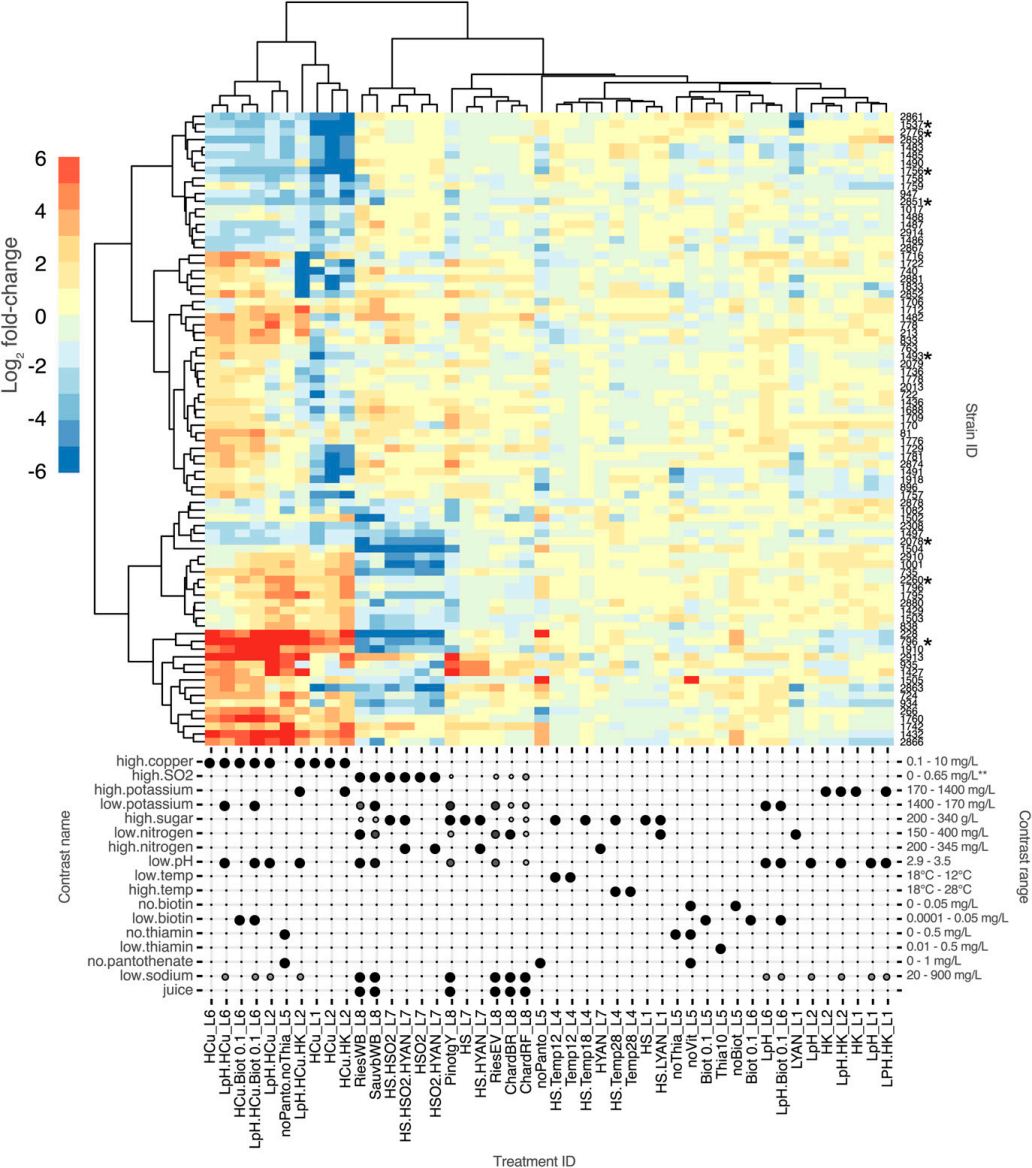
Strain fitness (log_2_ fold-change) clustered according to strain across treatment conditions (Y axis) and treament according to strain performance (X axis). Treatment fitness values show changes over time relative to the changes observed in a reference condition. Specific treatment factors are indicated in the balloon plot below the heat map. Spot size and fill is mapped to the difference between treatment and reference for any given compositional factor. The tested range of the factors is given on the right of the plot. SO_2_ contrast range is given as calculated molecular SO_2_. Detailed treatment descriptions are provided in File_S1-T5. Strain numbers refer to the source strains used to make the barcode collection. The culture collection ID, barcoded strain ID and common name are provided in File_S1-T1. Strains with an asterisk indicate those assessed in single inoculum experiments (Figure 5).

Once the robustness of the fitness profiling methodology had been established, serial competitive fitness experiments were initiated under a range of oenologically relevant conditions (Figure 4). The effect of 38 unique compositional conditions on strain competitive fitness were studied using a serial inoculation strategy (Figure 1C). The pairwise comparisons reported here span 2 serial inoculations (approximately 16 generations, File_S2 Figure S9). Evaluation of competitive fitness in reference conditions was achieved through comparing strain counts following two serial inoculations (Figure 1C, T3) to strain counts following initial growth in the reference medium (T1).

A pairwise comparison of fitness profiles in the reference media, representing 7 different experimental batches, showed a high correlation across reference conditions (r > 0.7) except for L4 *vs.* L7 (r = 0.609) (File_S2, Figure S10). The reference conditions were chosen to model a white juice with moderate sugar concentration (200 g/L), sufficient yeast assimilable nitrogen to support robust fermentation (400 mgN/L), low concentrations of trace elements relative to typical Chardonnay juice, no SO_2_ and moderate pH (3.5). In one experiment (L7) Chardonnay juice was used as the reference condition. Significant fitness variation between strains was observed in all reference conditions for many of the strains in this study (File_S2, Figure S10). Four strains performed particularly poorly (AWRI1427, 1482 and 2913), displaying significantly reduced abundance after two propagation cycles, not only in the defined medium but also in Chardonnay juice. Two of these strains are historical winery isolates and two are commercially available wine yeast recommended for white wine production.

Yeast strain fitness was assessed in a range of treatment conditions. By comparing changes in strain abundance in a treatment (Figure 1C, T3 and T1) relative to a reference condition over time, profiles of strain responses to variations in medium composition could be assessed. The different contrasts and the respective fold-change fitness estimates are shown as a heat map in Figure 4. The complete analysis from which the heat map is drawn is provided in File_S1-T7. The data reported here spans 7 independent experiments (batches) with contrasts made to the reference condition within each batch.

Variations to medium or juice compositional parameters were chosen based on previously identified ranges of those variables in Chardonnay juice (Hagen *et al.* 2008; Schmidt *et al.* 2011) or conditions to which wine yeast might be exposed during production. Variation in nitrogen assimilation capacity has been associated with yeast fitness (Ibstedt *et al.* 2015) and adaptability to different wine environments (Brice *et al.* 2018), SO_2_ is a known antimicrobial including against yeast (Divol *et al.* 2012), copper tolerance varies widely across winemaking strains (Brandolini *et al.* 2002; Cavazza *et al.* 2013) and thiamine limitations resulting from uncontrolled growth of organisms other than *S. cerevisiae* have been associated with poor fermentation outcomes (Bataillon *et al.* 1996). It was our aim to understand responses of a wide selection of wine yeast to these varied conditions as well as to understand whether different compositional variables interact to further limit the performance potential of individual wine yeast strains. A complete summary of the different experimental variables, the contrasts made, and medium/juice compositions explored in this work is provided in File_S1-T6.

#### Copper and sulfite:

Two conditions, 10 mg/L of copper and 15 mg/L free SO_2_ (0.5 mg/L molecular), had strong but unique strain specific fitness impacts and clustered independently of each other and other test variables. These two conditions were also dominant when tested in combination with other conditions. To a limited extent copper and sulfite tolerance appear to be mutually exclusive. Of the 24 strains with significantly increased fitness in 10 mg/L copper, 16 were significantly less fit in 15 mg/L free SO_2_ (66%) and of the 23 strains with significantly improved fitness in 15 mg/L free SO_2_, 12 were significantly less fit in high copper (52%). Only 2 strains were observed to have significantly increased fitness in both 10 mg/L copper and 15 mg/L free SO_2_.

Copper tolerance and sensitivity was evaluated in three independent experiments and although all treatments containing elevated copper concentrations clustered together, batch effects are evident through the formation of two sub-clusters within the larger ‘high copper’ treatment cluster. All the members of the second sub-cluster are derived from experiments L1 and L2. Low fitness scores of a subset of strains appears to drive the clustering behavior of these treatments. For example, the strain cluster containing AWRI1729, AWRI1781, AWRI2874, AWRI1491 and AWRI1918 all have reduced representation in HCu_L1 and HCu_L2 but not in HCu_L6. The base medium in experiments L1 and L2 has a low potassium concentration but the strain performance profile is not altered by the addition potassium (HCu.HK_L2). However, the lower pH in LpH.HCu_L2 resulted in a profile that clustered with HCu_L6, alleviating the reduced representation of the strains mentioned above. In general, these batch effects could not be accounted for by experimentally defined variations in medium composition and the source of the batch effects observed here remain to be resolved.

Yeast strains were designated as copper sensitive or tolerant if they showed significant deviation from the reference condition in at least two of the three ‘copper only’ contrasts. Twenty four percent of the strains evaluated in this study were found to be copper sensitive (significantly reduced mean fitness in copper-only contrasts). This high prevalence of copper sensitivity in commercial wine yeasts has been noted previously (Cavazza *et al.* 2013) and of the strains exhibiting the lowest fitness in high copper, AWRI1537 has been previously demonstrated to be inhibited by oenologically relevant copper concentrations (Ferreira *et al.* 2006). Resistance to copper in yeast has been shown to be mediated predominantly by the metallothionein Cup1 (Fogel and Welch 1982) with copy number expansion explaining 44.5% of phenotypic variation in copper tolerance (Peter *et al.* 2018). Substantial copy number diversity exists among wine yeasts ranging from 0 – 14 copies per strain (Steenwyk and Rokas 2017) although higher copy numbers have been shown to be possible without substantial fitness costs but also without greater enhancement to copper tolerance (Thierry *et al.* 2016).

SO_2_ sensitivity was also evident in a similar proportion of the strain set, with 32% of strains showing a reduction in abundance in medium containing 15 mg/L free SO_2_ (significantly reduced mean fitness in SO_2_-only contrasts). In grape juice and wine, SO_2_ exists in several forms, with the most biologically inhibitory being the neutral, volatile form known as molecular SO_2_. The prevalence of this form in grape juice is pH dependent with its abundance ranging 6 fold between pH 3.0 and pH 3.5 (Waterhouse *et al.* 2016). Conditions in which calculated molecular SO_2_ is highest, rather than concentrations of free or total SO_2_, cluster together (Figure 4, treatments RiesWB_L8, SauvbWB_L8, HS.HSO2.HYAN_L7, HSO_2__L7, HSO_2_.HYAN_L7). Of the six strains most sensitive to SO_2_, three were winery isolates (AWRI228:Log_2_FC -8.3, AWRI1001: Log_2_FC = -4.9, AWRI735: Log_2_FC = -4.8), one was a non-commercial interspecific hybrid (AWRI1504:Log_2_FC = -8.1) and two were commercial strains (AWRI2863:Log_2_FC -5.7, AWRI2078: Log_2_FC = -4.7).

Of the two sulfite sensitive commercial strains, AWRI2078 has previously been associated with SO_2_ sensitivity (Aranda *et al.* 2006) and neither are known to harbor the *SSU1-R* locus. The most SO_2_ sensitive strain in the work by Aranda *et al.* (2006) did not feature in the SO_2_ sensitive group in this study (AWRI1493). SO_2_ tolerance in *S. cerevisiae* is mediated by the efflux pump Ssu1 (Park and Bakalinsky 2000) but unlike the mechanism that gives rise to copper tolerance, variation in SO_2_ tolerance in wine strains of *S. cerevisiae* is predominantly the result of reciprocal chromosomal translocations between chromosomes VIII and XVI (Goto-Yamamoto *et al.* 1998; Pérez-Ortín *et al.* 2002) although another translocation has recently been identified (García-Ríos *et al.* 2019). These translocations broadly result in the coding regions of *SSU1* now being driven by the more highly expressed promoter of the *ECM34* gene, although substantial heterogeneity exists in the precise structure of the translocated promoter, and therefore overall expression of *SSU1* and subsequent SO_2_ tolerance (Yuasa *et al.* 2004; 2005). However, AWRI1493 (71B) does not have this translocation and instead it has been shown that SO_2_ is an inducer of *SSU1* in 71B (Nardi *et al.* 2010), whereas *SSU1* appears to be expressed constitutively in strains carrying the translocation (Nardi *et al.* 2010). Induction of *SSU1* over the course of serial inoculations therefore likely explains the neutral fitness score of 71B (AWRI1493) in this work.

#### Nitrogen:

In grape juice fermentations nitrogen can be the primary limiting nutrient, ranging in concentration from less than 100 mg/L yeast assimilable nitrogen (YAN) to more than 500 mg/L (Schmidt *et al.* 2011). Its concentration in grape juice is determined by both vineyard conditions and winemaker intervention and concentrations of 150 mg/L are considered the minimum required for successful completion of fermentation (Bell and Henschke 2005). The relative balance of ammonia and amino acids as well as their absolute concentrations affect yeast transcriptional activity (Godard *et al.* 2007) and growth (Gutiérrez *et al.* 2013) providing the building blocks of biomass that is key to robust fermentation kinetics (Varela *et al.* 2004). Strain specific differences in nitrogen demand (Julien *et al.* 2000) and amino acid utilization capability (Gutiérrez *et al.* 2013) have previously been observed. Strains having higher nitrogen demand are able to support higher growth rates (Gutiérrez *et al.* 2012). Gutiérrez *et al.* (2012) calculated that the strain with the highest nitrogen demand of the four that were studied would have the greatest fitness advantage in all nitrogen concentrations above 40 mgN/L, suggesting that strain specific nitrogen demand is a driver of fitness.

The effect of nitrogen on competitive fitness was evaluated in two ways: either by comparing strain abundance in nitrogen supplemented juice (YAN 345) with non-supplemented juice (YAN 200, reference condition) or by comparing strain abundance in low nitrogen defined medium (YAN 150) to high nitrogen defined medium (YAN 400, reference condition). Minor changes in fitness were observed when evaluating the effect of low nitrogen juice supplementation. The strains AWRI1082 (log_2_FC = 2.0), AWRI1910 (log_2_FC = 1.5) and AWRI1502 (log_2_FC = 1.2) showed higher fitness under nitrogen supplementation, increasing their representation in higher nitrogen conditions.

More prominent fitness differences were observed when transferring strains from nitrogen sufficient to nitrogen deficient conditions with the strains AWRI1537 (log_2_FC = -5.5), AWRI2863 (log_2_FC = -4.5), AWRI2861 (log_2_FC = -4.3), AWRI1756 (log_2_FC = -3.5), AWRI1781 (log_2_FC = -3.4), AWRI1483 (log_2_FC = -3.1) and AWRI2881 (log_2_FC = -3.0) all exhibiting decreased fitness in the low nitrogen medium. Two of the poorest performing strains, AWRI1537 and AWRI2861, have both been reported to have low and high nitrogen demand respectively (supplier technical data sheet). Of the better performers in low nitrogen defined medium AWRI2260 (log_2_FC = 1.2), AWRI2880 (log_2_FC = 0.9) and AWRI1429 (log_2_FC = 0.9) are reported to have either low or average nitrogen demands. AWRI1537 marginally improved its representation in nitrogen supplemented juice although initial nitrogen status of the juice (YAN = 200 mgN/L), while less than ideal, may be considered sufficient. In this data set we therefore find little support for the idea that strain specific nitrogen demand drives a fitness advantage in *S. cerevisiae* wine yeast strains.

#### Vitamins:

The defined medium used in this work contains 9 vitamins (thiamine, riboflavin, pyroxidine, pantothenate, nicotinic acid, myo-inositol, biotin, folate and 4-amino benzoic acid). In the absence of any vitamins (except myo-inositol) a single strain, AWRI1505, was able to substantially increase its representation in the mixed population (log_2_-fold change = 7.3, File_S1-T7) compared to the reference condition. AWRI1505 is an interspecific hybrid of *S. cerevisiae* and *S. uvarum* (Borneman *et al.* 2016). *S. uvarum* is historically associated with biotin proficiency (Oura and Suomalainen 1982) and AWRI1505 exhibits a moderate fitness advantage in the absence of only biotin (log_2_-fold change = 2.9). Although previous work suggested that biotin concentrations of less than 1 μg/L may be limiting, especially in nitrogen sufficient conditions (Bohlscheid *et al.* 2007), in the work presented here addition of 0.1 μg/L of biotin was sufficient to return differential strain fitness (File_S1-T7) but not biomass formation (File_S2, Figure S4E) to that of the reference condition. Together these data indicate that biotin prototrophy is only a partial driver of the AWRI1505 fitness gain in the absence of vitamins. Surprisingly addition of only biotin at 5 μg/L to the vitamin-free condition generated a differential strain fitness profile that clustered with the 10 mg/L copper treatments (Figure 4, L5_noPanto.noThia). Thiamine has previously been shown to play a supporting role in the maintenance of intracellular redox state (Wolak *et al.* 2014), and oxidation of the Mac1 transcription factor has been shown to both respond to intracellular redox changes and play a role in copper sensing (Dong *et al.* 2013). Furthermore, iron/sulfur complexes that are key intracellular copper targets, are also sensitive to reactive oxygen species (Vallières *et al.* 2017). Therefore, the absence of specific vitamins could mimic a high copper fitness profile via oxidative phenomena. However, the absence of thiamine or pantothenate alone did not mimic the high copper concentration fitness profile suggesting other vitamins, or combinations thereof, give rise to the ‘high copper-like’ fitness profile.

In contrast to the moderate fitness advantage in the absence of biotin, AWRI1505 exhibited a large fitness advantage in the absence of pantothenate (log_2_-fold change = 7.6) which is similar to its advantage in the vitamin-free condition. Only one other strain, AWRI228, exhibited such a large fitness advantage in the absence of pantothenate (log_2_-fold change = 10.8) but this strain had a neutral fitness profile in the vitamin-free conditions. Unlike AWRI1505, AWRI228 is not an interspecific hybrid but represents a historical isolate of *S. cerevisiae* deposited with the AWRI culture collection in 1949. *S. cerevisiae* has been shown to be capable of pantothenate production, mediated by the amine oxidase Fms1 (White *et al.* 2001) and aldehyde dehydrogenases Ald2 and Ald3 (White *et al.* 2003), and strain dependent differences in pantothenate requirements have been demonstrated (Wang *et al.* 2003). However, despite the existence of sequence variation in *FMS1*, no associations between those variations and strain fitness in the absence of pantothenate were identified.

In this work we show that variation in pantothenate requirements exist across a wide range of wine yeast strains. Enologically, pantothenate has been shown to interact with nitrogen availability to drive aroma compound production in wine yeast (Bohlscheid *et al.* 2011) albeit at concentrations lower than those typically found in grape must (Hall *et al.* 1956; Hagen *et al.* 2008). This suggests that yeast strain variation in pantothenate production capacity has the potential to impact fermentation outcomes beyond performance.

The impacts of other conditions such as temperature (12°, 17° and 28°), high sugar concentration (280 g/L), low pH (pH 3.0), and nitrogen availability were less pronounced than either high copper or SO_2_ concentrations.

Osmotic stress related to high sugar concentration has been shown to induce strain dependent variation in lag phase of up to 4 hr at 28° and up to 15 hr at 16° (Ferreira *et al.* 2017). Such strain specific effects were not evident in defined medium contrasts of 200 g/L and 280 g/L sugar concentrations. Contrasts undertaken in Chardonnay and Pinot gris juice (200 g/L *vs.* 280 g/L and 343 g/L respectively) revealed two strains, AWRI935 and 1427, that were observed to increase their abundance in high sugar conditions (log_2_-fold change = 4.0 and 4.1). These strains were two of the poorest performers in the reference conditions (File_S2, Figure S10), reducing their representation in the mixed population by up to 30-fold.

Similarly, contrasts between strain growth at pH 3.0 with pH 3.5, did not reveal substantial strain specific effects either in defined medium or grape juice, with or without potassium supplementation. pH-related, strain-specific impediments to both growth rate and biomass formation have previously been observed in both defined medium and grape juice (Kudo *et al.* 1998; Schmidt *et al.* 2011). It was therefore surprising not to see greater pH-related fitness variation revealed within the context of a competitive experimental format.

It was of interest to explore whether different compositional elements could interact to either exacerbate or alleviate strain specific responses, however, no interactions were detected. For the combinations that contained copper, or SO_2_, the fitness profile of the combined treatments clustered with either high copper or high SO_2_ alone. Some combinations of conditions were excessively harsh resulting in substantial suppression of biomass formation. These conditions were most notably, absence of vitamins, pH 3.0 and 10 mg/L copper, 0.1 mg/L biotin and 10 mg/L copper, and 280 g/L of sugar fermented at 12° (File_S2, Figures S4B, S5G, S5E and S3D). It is likely that conditions in which growth is reduced to such an extent limit the opportunity for fitter individuals to increase their representation within the mixed population, thereby limiting the resolution of such contrasts.

### Comparison of pooled inoculum fitness estimates with single inoculum fermentation performance

**Figure 5:**
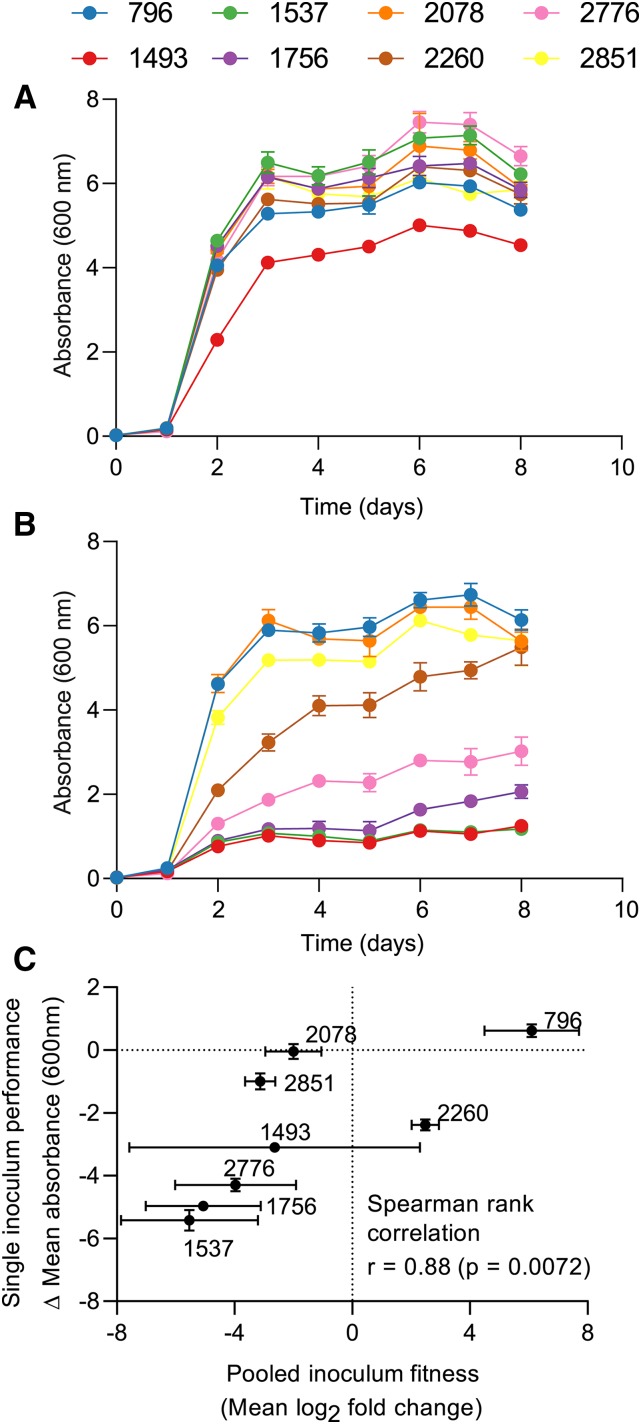
Comparison of growth in single inoculum fermentations of eight wine yeast strains in 0.035 mg/L (A) and 10 mg/L (B) of copper. Graphs show mean absorbance measured at 600 nm (± SD, n = 3). C) Differential growth in single inoculum fermentations compared to mean pooled inoculum fitness (copper only contrasts). Difference in single inoculum growth is shown as the difference in mean absorbance between a high copper condition and reference defined medium (∆ abs = abs HCu – abs Ref). The measurements were taken on day 3 post inoculation. Error bars indicate standard deviation (n = 3).

The relationship between competitive fitness estimates derived through pooled inoculum experiments and traditional performance estimates obtained through single inoculum fermentations was explored using differential growth in low (0.035 mg/L) and high (10 mg/L) copper concentrations as a discriminatory and industrially relevant compositional variable. Eight individually inoculated strains were evaluated. Figure 5A shows the biomass accumulation of these strains in low copper containing medium demonstrating minimal growth-based differences between them in the low copper condition, except for strain AWRI1493. Figure 5B highlights substantial differences between strains in growth rate (change in absorbance at 600 nm over time) and final biomass formation (as indicated by their maximal absorbances at 600 nm) in the presence of 10 mg/L of copper.

The relationship between differential biomass formation in single inoculum experiments (abs day 3 (high copper) – abs day3 (low copper)) and competitive fitness scores (mean log_2_ fold-change from three independent 10 mg/L copper contrasts) is shown in Figure 5C. For the majority of strains (*i.e.*, AWRI796, AWRI2851, AWRI1493, AWRI2776, AWRI1756 and AWRI1537) differential growth in single inoculum conditions was consistent with competitive fitness scores. Strain AWRI1493 alone exhibited a great deal of variation across the independent evaluations of fitness. Two strains consistently showed an inverse relationship between single inoculum performance and pooled inoculum fitness. Strain AWRI2260 was determined to be copper tolerant in 3 independent competitive fitness experiments (mean log_2_ fold-change = 2.5 ± 0.48) but consistently showed an inhibited growth rate in single inoculum evaluations of performance. Conversely, strain AWRI2078 was determined to be copper sensitive in competitive fitness evaluations (mean log_2_ fold-change = -2.03 ± 0.95) but was copper resistant in single inoculum cultures. Understanding the basis for this contradiction requires further work.

## Conclusion

Barcode sequencing of pooled wine yeast strains is demonstrated to be a valuable tool in the phenotyping of industrial yeast. It allows performance characteristics to be assessed in both grape juice-like model media and grape juice itself and complements parallel phenotyping tools such as single inoculum micro-well fermentations and solid agar growth assays. It has the advantage over other forms of phenotypic profiling methods in that wine yeast performance can be assessed in non-sterile conditions that may not lend themselves to plating or optical density-based methods of enumeration, such as in high solids wine fermentations. The barcoded collection permits an in-depth characterization of a wide diversity of industrial yeasts, adding to the growing body of tools that are available for the study of yeasts in general.

A major finding of this work was that two dominant discriminators of *S. cerevisiae* wine yeast strains, copper and SO_2_ tolerance, appear to be mutually exclusive phenotypes in a substantial proportion of the strains evaluated. Without an apparent link between the two modes of delivering tolerance to either stress a larger number of strains tolerant to both conditions was expected. The relationship between copper and SO_2_ tolerance will be examined in more detail in future work.

In wine research, the majority of strain evaluation is undertaken using single inoculum fermentation of medium sterilized by filtration. Sterilization ensures that the organism that is inoculated is the one that is measured in subsequent analyses. However, in practice wine making yeasts need to compete with the rich microbial ecology that constitutes a freshly prepared juice in addition to the vast array of compositional variables that they might encounter. In addition, filtering not only sterilizes the starting material but facilitates the estimation of biomass accumulation through the measurement of absorbance by removing particulate material. Those particulates, and the sterols that are also removed by filtration, have both been shown to be important contributors to yeast growth and fermentation performance under enological conditions (Delfini 1993; Delfini and Costa 1993; Casalta *et al.* 2019). A requirement for filtering also precludes the use of more complex matrices such as red grape juice, the filtering of which removes much of the phenolic material important for understanding that particular winemaking condition. Using a barcoded strain set circumvents these limitations and enables the parallel evaluation of many strains under identical conditions. It provides a complimentary approach, enabling the assessment of strain performance in matrices that might be precluded from standard methods of evaluation.

Differences were observed in the performance of two out of eight strains in single inoculum fermentations compared to the performance of the same strains in pooled inoculum fermentations. This raises interesting questions about the interplay between organisms in competitive environments and the degree to which such results can be generalized and perhaps more importantly, how laboratory evaluations can be extrapolated to the winery. Nevertheless, having the capacity to evaluate strain performance using a range of approaches will help build a more comprehensive and robust view of a strains industrial capability and having the ability to evaluate larger numbers of genetically diverse yeast will help to enrich our understanding of yeast biology.
